# Design of a New Sensor for Determination of the Effects of Tractor Field Usage in Southern Spain: Soil Sinkage and Alterations in the Cone Index and Dry Bulk Density

**DOI:** 10.3390/s121013480

**Published:** 2012-10-08

**Authors:** Diego L. Valera, Jesús Gil, Juan Agüera

**Affiliations:** 1 Department of Engineering, University of Almería, Ctra. Sacramento s/n, 04120 Almería, Spain; 2 Department of Rural Engineering, University of Córdoba, Campus de Rabanales, Edificio Leonardo da Vinci, Carretera Nacional Nacional IV-A km 376, 14014 Córdoba, Spain; E-Mails: gilribes@uco.es (J.G.); jaguera@uco.es (J.A.)

**Keywords:** soil compaction, trafficability, profile meters, rut depth, tractor traffic

## Abstract

Variations in sinkage and cone index are of crucial importance when planning fieldwork, and for determining the trafficability of farm machinery. Many studies have highlighted the link between higher values of these parameters and dramatic decreases in crop yield. Variations in the dry bulk density and cone index of clayey soil in Southern Spain were measured following each of five successive passes over the same land with the three types of tractor most widely used in the area (tracked, two-wheel drive and four-wheel drive). In addition, sinkage (rut depth) of the running gear was measured using a laser microrelief profile meter. This device, which integrates three sensors, was specifically designed for these experiments, as was an electrical penetrometer to determine the cone index, and both instruments proved reliable and accurate in the field. The main goal of this study was to design, manufacture and test these new devices. The first pass caused most soil alteration when compared to successive passes for all types of tractor tested and soil conditions prevailing during the tests. (Heavier) four-wheel drive tractors were found to cause greater soil damage (sinkage, cone index and dry bulk density) than two-wheel drive and track tractors. There was no statistically significant difference between the two latter types. The greatest alterations were recorded in the top 10 cm of the soil. The results show that soil compaction should be avoided as much as possible. This can be achieved by ensuring that tractors always travel along the same tracks, especially in the wet season. At present these aspects are not considered by farmers in this area.

## 1. Introduction

Soil compaction is related to dynamic properties that change with time due to various crop operations and environmental factors. Measuring soil compaction is usually a tedious, time consuming and expensive task. One approach frequently taken to investigate the variability in physical properties of the soil is to measure soil strength indices [1].

Since no single or direct measurement exists for expressing the degree of soil compaction, it can only be determined indirectly by measuring a range of different soil properties [2,3]. Soil conservation during farm machinery trafficking basically depends on soil texture, organic matter and soil water content [4,5].

Soil moisture content is the most important factor in the compaction process [6], but farmers often cannot easily control soil moisture during farm machinery trafficking [5]. The most obvious visual indicator of topsoil compaction is the rut depth left by agricultural tractor and machinery on the soil [7]. This depth is principally related to the initial soil condition, tyre pressure, tyre width and number of passes [8]. On the other hand, topsoil damage is related to ground pressure, while subsoil damage is related to the total axle load of the agricultural machinery [9].

The commonly used parameters for measuring levels of soil compaction include cone index [4,7–9], soil sinkage [6] and dry bulk density [2,3,5]. Compaction of cultivated soil has significant economic and ecological impacts, affecting root growth, seed emergence and plant establishment [10,11]. The economic implications are difficult to quantify, given that the precise effects of compaction are not clearly defined and are related to former soil conditions, soil management, crop development and weather conditions [9,12].

Economic losses due to soil compaction derive directly from the decrease in crop yield and indirectly from non-optimal use of soil resources and failure to exploit soil improvements, as well as from the loss of arable land due to greater erosion.

The environmental impact of soil compaction may be related to the increased erosion [13], transportation of agricultural chemicals and emission of gases likely to cause climatic changes. Increased anaerobiosis leads to higher ground emission of CH_4_ and N_2_O [14]. Nitrogen loss due to denitrification is normally greater in compacted soil than in non-compacted soil [15].

The object of this research was to measure soil alteration caused by the most widely used tractors in Southern Spain, while at the same time designing, developing and field-testing the equipment required, a new device, which integrates three sensors. These tools have made it possible to establish criteria to reduce soil compaction caused by agricultural machinery, thus minimising the possible economic and environmental losses.

The following section presents the developed equipment. Then the field test and the procedures that were carried out are described, including the tractor descriptions and data analysis. In the Results and Discussion section, we analyze the effects of the passage of each type of tractor on the sinkage, cone index and dry bulk density.

## 2. Materials and Methods

### 2.1. Development of the Electrical Penetrometer

**Figure 1.**
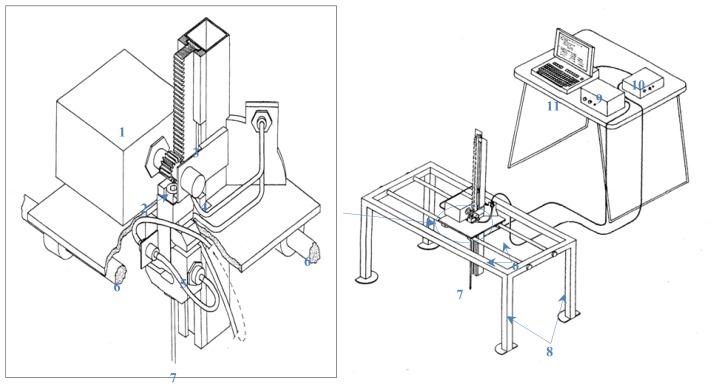
Electrical soil cone penetrometer. (1) Electric motor, (2) pinion, (3) zip, (4) potentiometer, (5) load cell, (6) guides, (7) driving shaft, (8) frame, (9) control unit of speed and direction of rotation, (10) signal conditioner, (11) PC.

Several researchers have conducted studies aimed at measuring the soil compaction profile, summarising and discussing previous approaches [1,16]. The soil cone penetrometer is recommended as a measuring device to provide a standard uniform method of characterising the penetration resistance of soils. The force required to press the 30-degree circular cone through the soil, expressed in kilopascals, is an index of soil strength called the cone index [17]. An electrical penetrometer (Figure 1) was designed for measuring penetration resistance, recording cone index values up to a maximum depth of 30 cm at 5 mm intervals, according to ASABE Standards [17,18]. This maximum depth was selected as preliminary tests suggested that any soil deformation was confined to the top 30 cm of the profile [16]. The electrical penetrometer reduces possible sources of measurement error, since both the penetration speed and the separation between penetrations are constant.

The frame provides support for the sensors, motor, pinion, zip and driving shaft. The measuring device runs along two rails which are marked to allow precise distancing between penetrations; in this case measurements were taken every 7.5 cm. The load cell used has a 1000 N FS with an overrange of 150% (Mutronic S.A., Madrid, Spain). The potentiometer (Spectrol 534, Spectrol Electronics Corporations, Ontario, CA, USA) detemines the depth of the base of the cone at the tip of the driving shaft, which is powered by the electric motor (Automat 80.600, Automat Industrial S.L., Granollers, Spain; 50 W). In order to obtain cone index values, the *ad hoc* “ES” software was developed. This software manages the recording of data according to ASABE Standards [17,18]. The penetrometer functions at a constant rate of penetration and it allows accurate evaluation of the distance between penetrations; it too is a sturdy low-cost device which is specially adapted for studying soil compaction caused by tractors.

### 2.2. Design of the Microrelief Profile Meter

The Microrelief Profile Meter must provide accurate measurements of a set of point coordinates, data collection speed, field portability, structural stability and system reliability. It is a non-contact device which allows a high number of sampling points, 3D plots and rapid assessment of results. At the same time, its small size means that it is easy to transport, and it also proves sturdy enough for use in the field. The specific design criteria were as follows: to obtain elevation measurements with an accuracy of 50 μm; to collect data up to a maximum of 14,000 points (x,y,z, coordinates) within 1 m^2^ of the trial surface without coming into contact with the soil; to allow movement by two people; and to ensure that the system is durable and reliable during field applications. Several authors have pointed out the suitability of similar design criteria. Several authors have summarized previous approaches for measuring soil surface topography [16,19–23], which include pin displacement units, height-transducer probes, and several non-contact optical systems using lasers [19].

**Figure 2.**
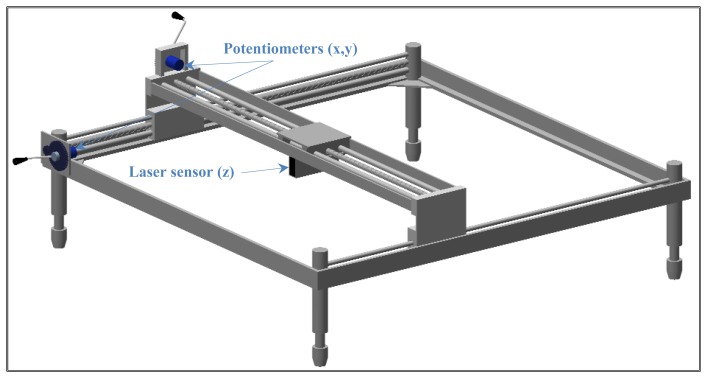
Laser microrelief profile meter for field studies of soil compaction.

The measurement system comprises three sensors: two potentiometers to measure the “x” and “y” axes (3501 Precision Potentiometer, Bourns, Riverside, CA, USA) and a laser displacement sensor to measure the “z” axis to a resolution of 50 μm (LB-301, Keyence, Osaka, Japan). The system is equipped with a laser signal controller, 12-bit analog-digital converter (DI-220, DATAQ Instruments, Inc, Akron, OH, USA), power supply and a portable PC to store, process and display soil surface data. The converter is portable, and is configured via software to allow both analog and digital input and output. It is connected to the PC through a parallel port, and uses a rechargeable battery. Figure 2 displays a 3-D rendering of the design.

**Figure 3.**
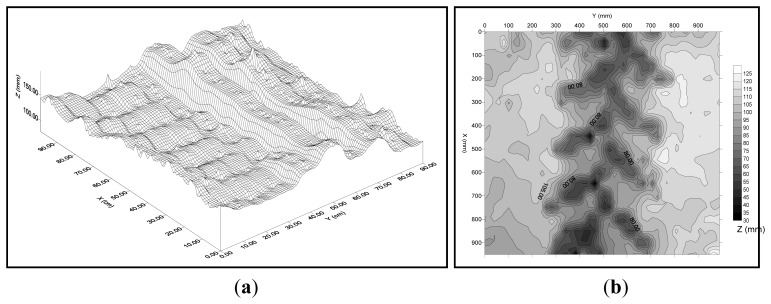
3D plot of tracked tractor traffic Fiat 82-85 M (**a**) and 2D plot of wheeled tractor traffic E-8110-DT (**b**).

This device allows extremely accurate 2D and 3D representation of the soil surface (Figure 3). Other microrelief meters allow more points of measurement [20] but take substantially longer to process the data.

The accuracy of all the instruments was tested in the metrology laboratory of the “Mechanization and Rural Technology” Research Group at Córdoba University (Córdoba, Spain), even though all sensors came with the manufacturers' calibration certificate. In addition, a 12-bit analog-digital converter was used to minimise loss of resolution. We believe that the accuracy of the instruments developed is more than sufficient for the analysing agricultural soil compaction. These novel devices were also compared with others that had been developed by the above-mentioned research group, and were found to improve upon the performance in the field of the earlier models. These tools have made it possible to establish criteria to reduce soil compaction caused by agricultural machinery, thus minimising the possible economic and environmental losses.

### 2.3. Field Test

The study site (Latitude 37°24′N; Longitude 5°35′E) was located at the “Tomejil” Experimental Farm in the Guadalquivir Valley region of Seville (Southern Spain), which is at 76 m a.s.l., with a typical Mediterranean climate with oceanic influences. The mean annual precipitation and temperature are 700 mm and 18.6 °C, respectively. Field experiments were conducted over three years and carried out during the normal drying cycle of the soil. The terrain in this area (Table 1) comprises clayey soil on Miocene beds, defined under the USDA classification system as Entic Peloxererts.

Most of the clayey fraction consists of montmorillonite (70%), with 20% ilite and 10% kaolinitic with chlorite. Due to its high content of expanding clay minerals, frequent intra-profile movements have prevented the formation of differentiated horizons. Soils in this area are generally deep. Weathered materials are only found at higher elevations due to the entrainment of soil into basins, which in the rainy season often leads to water logging. Relief is not highly pronounced and is characterised by smooth undulations with slopes of between 1 and 5%.

The following procedures were carried out:

1. Measurements (soil surface (laser microrelief profile meter), initial cone index (electrical penetrometer), initial dry bulk density and humidity (sampling device)) were taken in untrafficked soil, that is before tractors had passed over the surface.
2. A tractor was driven over the same area in which the first measurements had been taken, and these were repeated.
3. The process was repeated four more times, for a total of five passes over the surfaces in which the unaltered soil parameters had been recorded initially. This number of passes was selected in order to simulate the typical traffic intensity in the region under study.
4. The process was repeated for each tractor type. These tractors were models that are frequently used on commercial farms in the experimental area.
5. For each day that a tractor was tested, this meant: 198 tests with the penetrometer (12,078 C.I. values), six tests with the microrelief profile meter (12,000 co-ordinates) and 24 soil samples.

In order to calculate dry bulk density and humidity, a prototype sampling device was designed to allow soil samples to be extracted with as little alteration of the soil as possible. The mechanism comprised a cylindrical support frame and a sampling cylinder measuring 81 mm in height and 47.5 mm in diameter (143 cm^3^ in volume). Handles were fitted at one end of the frame to facilitate embedding of the lubricated sampling cylinder, which was fitted to the lower end of the frame to collect the soil sample. Once the sampling cylinder had been embedded in the soil, the whole device was withdrawn and the full sampling cylinder was removed from the cylindrical frame. The soil sample was then immediately inserted in a plastic bag, weighed and placed in the oven. A new sampling cylinder was then fitted to the frame, and the operation was repeated. In order to carry out successive soil tests, 100 sampling cylinders were used with the same cylindrical support frame. Both the cylindrical frame and the sampling cylinder were made of stainless steel.

The device described above was used to obtain four samples both at the surface and at a depth of 30 cm, before trials and after each of the five passes made by the three types of tractor. All samples were obtained from tractor ruts, except for the first batch of samples which were obtained from soil prior to the passing of tractors. The moisture content and dry bulk density of each sample were calculated.

### 2.4. Tractor Descriptions

The three most widely-used tractors in the area [16] were studied in this work: a mid-heavy weight four-wheel-drive Ebro E-8110-DT, a tracked Fiat 82-85 M vehicle and a mid-range two-wheel drive John Deere JD1840 model. The aim of this work was to quantify the damage caused to the soil by the main tractor types used in the Guadalquivir Valley, irrespective of their different characteristics, since agriculture is a key social and economic sector in this region. Table 2 summarises the most relevant technical features of the two types of tractor tyres used.

The features of the Fiat 82-85 M tracked vehicle were as follows: power 58.8 kW, weight 44492 N and running gear comprising two 37-plate tracks, with a track-wheel diameter of 0.625 m and a track- plate width of 0.4 m.

### 2.5. Data Analysis

Tractor sinkagewas measured as rut depth using a laser microrelief profile meter. Sinkage was measured as the volumetric difference between initial soil surface (soil prior to vehicle passes) and soil surface after each tractor pass. This parameter is widely used in studies of soil compaction [24–26].

For the 3D representation of the sampled soil surface, the Surfer 5.0 programme was used (Golden Software Inc., Golden, CO, USA). This programme also allowed calculation of sinkage, expressed as the volumen, after each tractor pass.

Dry bulk density was calculated with a Microsoft EXCEL spreadsheet using the standard procedure based on weight difference of a known sample volume before and after drying actual soil samples. The same programme was also used to calculate and plot the mean IC-Depth curves corresponding to each pass and the Dry bulk density-Pass, and Sinkage-Pass curves. Soil resistance to penetration was represented using the cone index [17,18].

All the statistical analyses [one-way and multifactor ANOVA, multiple range test, Fisher's least significant difference (LSD) procedure, variance check and box and whisker plots] were undertaken with Statgraphics Plus ver. 4.1 (Manugistics, Inc., Rockville, MD, USA).

Some of the statistical analyses carried out did not fulfil the basic hypotheses to allow the use of parametric statistics, and so the non-parametric alternative was used. The non-parametric techniques available have a much smaller range of applications than parametric techniques, since they are much less sensitive in detecting significant effects. Using these non-parametric procedures we found that the different parameters influenced the response variable, but it was necessary to use graphic procedures in order to obtain homogeneous groups among the factors (treatments).

## 3. Results and Discussion

For all tractor types tested, dry bulk density at the surface increased with each pass, becoming asymptotic by the third pass (Table 3). In all cases, the greatest quantitative increase was recorded between initial density and after the first pass.

**Figure 4.**
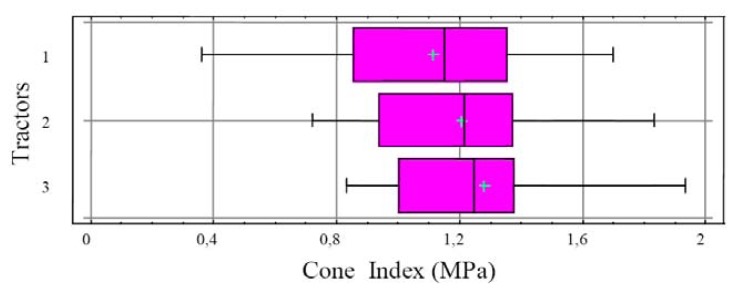
Box and Whisker Plot, Tractors type *vs.* Cone Index.

Nevertheless, mean dry bulk density at a depth of 30 cm was not affected by the number of passes or the type of tractor involved. No statistically significant differences were observed in penetration resistance with the three types of tractor (Figure 4).

Successive passes over the same surface caused a significant alteration in soil penetration resistance beginning with the first pass. However, no statistically significant differences were recorded for penetration resistance after the first, second, third, fourth and fifth passes (Table 4), that is, all the tractors in the trial produced the maximum alteration in this type of soil during the first pass.

The results show that soil compaction should be avoided as much as possible. This can be achieved by ensuring that tractors always pass along the same tracks, especially in the wet season. At present these aspects are not considered by farmers in this area.

**Figure 5.**
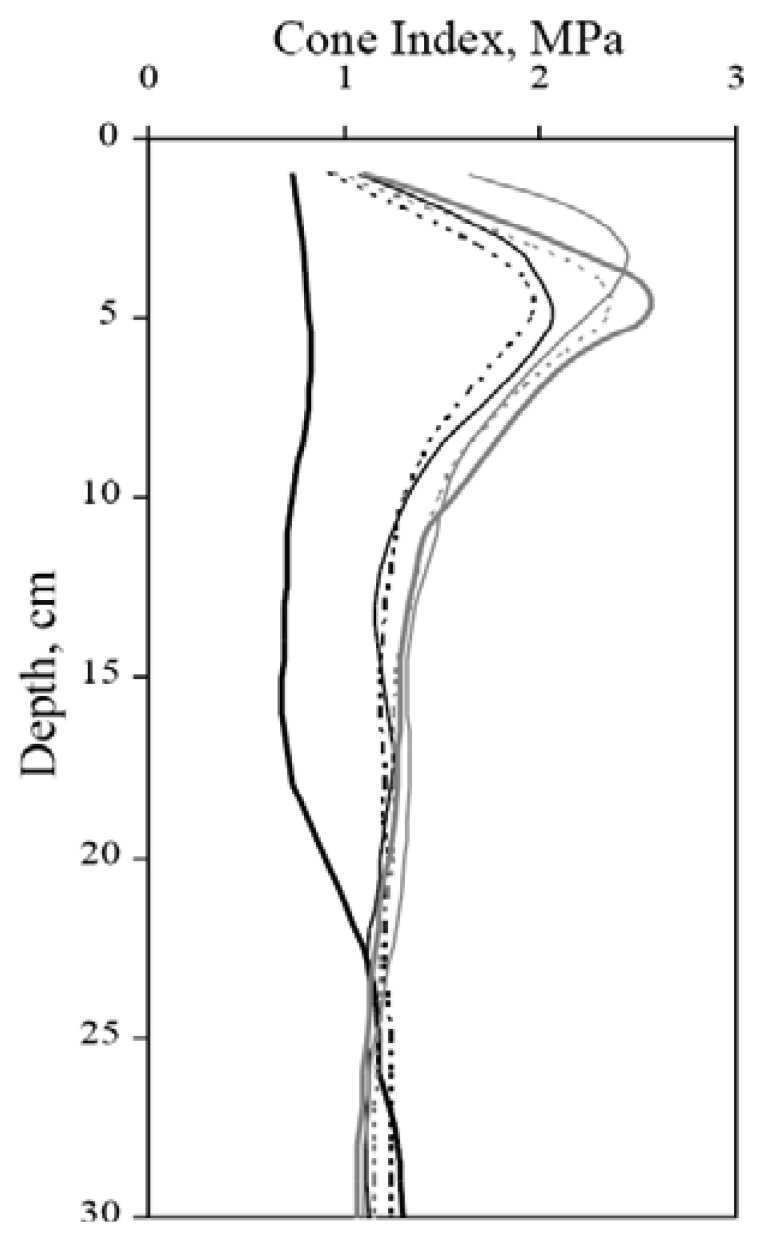
Evolution of penetration resistance by number of passes. ( 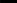) Initial, (---) 1pass, (─) 2 passes, ( 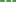) 3 passes, ( 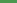) 4 passes, ( 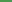) 5 passes.

The fact that the alterations in penetration resistance (cone index) were observed in the top 10 cm of soil (Figure 5) is worth highlighting. This result does not agree with that of Botta *et al.* [5], probably due to the substantial difference in the soil type. In the present study, the heavy soils with a high clay content of the Guadalquivir Valley (Spain) could explain the results obtained.

These results indicate that on such soils it would be advisable to adopt controlled traffic measures. In other words, farmers should ensure that farm machinery runs over the same tracks in which the soil has already been compacted. In this way they would avoid compacting the rest of the surface, as the greatest compaction and soil damage occur on the first pass. This result is confirmed for all of the parameters studied, and so by adopting this simple measure economic loss and environmental damage could be avoided. It should be pointed out that the economy of this area of Southern Spain relies to a great degree on agriculture and so the savings could be considerable.

**Figure 6.**
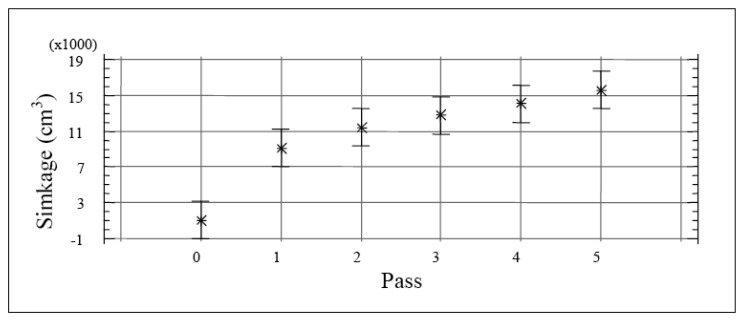
Means and 95% LSD Intervals, sinkage *vs.* pass number.

Greater sinkage occurred during the first pass, with significant increases in the third and fifth passes (Figure 6). The sinkage of the tractors increased at greater moisture contents. This result is in agreement with several previous studies, which also related sinkage to increased organic content of the soil [5].

For all of the tractors and soil conditions studied here, the first pass provided the greatest increase in dry bulk density, resistance to penetration and sinkage, compared to the second to fifth passes. There is a strongly positive relationship between tractor passes and cone index and the heavy tractor produced higher cone index values than the light tractors; this result is in agreement with Botta *et al.* [7].

The four-wheel-drive tractor produced the greatest increase in dry bulk density, resistance to penetration and sinkage, when compared to the tracked tractor and the two-wheel-drive tractor. No statistically significant differences were observed between the latter models. This may be accounted for by the weight/load-bearing surface ratios of each tractor.

## 4. Conclusions

Based on the material presented in this article and within the limits of our experimental conditions, several conclusions were reached. The design and development of the microrelief profile meter and electrical penetrometer have proven successful. The first vehicle pass was proved to cause most soil alteration when compared to the second through fifth passes, and this was true for all types of tractor and whatever the soil condition. The double traction tractor produced greater soil damage than both single traction and track tractors. There was no statistically significant difference between the two latter types. The greatest soil alteration was recorded in the top 10 cm of the soil.

Soil compaction should be avoided as much as possible by the farmer, especially in the wet season. This can be achieved by running farm machinery over the same tracks, although to date this has not been taken into account in the area of study, despite its great economic and environmental importance.

## Figures and Tables

**Table 1.** Some physical properties of the soil.

**Depth (cm)**	**Clay (%)**	**Silt (%)**	**Sand (%)**
0–23	70.5	29.4	0.1
23–84	72.6	26.3	1.0
84–122	73.4	23.5	3.1
122–150	70.2	28.9	0.9

**Table 2.** Characteristics of tractor tyres.

			**Front Wheels**			**Rear Wheels**		
	
**Model**	**Power** **(kW)**	**Weight** **(N)**	**Tyres**	**Tyre** **Pressure** **(bar)**	**Effective** **Diameter** **(m)**	**Tyres**	**Tyre** **Pressure** **(bar)**	**Effective** **Diameter** **(m)**
JD1840	46	27710	7.5 × 16″	2.5	0.68	16.9 × 30″	1	1.20
E-8110-DT	76	57820	13.4R × 28″	1.4	1.26	18.4R × 38″	1.1	1.66

**Table 3.** Multiple Range Test for dry bulk density at the surface by pass of tractor.

**Method: 95% LSD**			
**Pass**	**Count**	**LS Mean**	**Homogeneous Groups**
0	21	1135.93	X
1	21	1245.32	X
2	21	1285.31	X
3	21	1287.18	X
4	21	1307.01	X
5	21	1320.83	X

**Table 4.** Multiple Range Test for cone index *vs.* pass.

**Method: 95% LSD**			
**Pass**	**Count**	**LS Mean**	**Homogeneous Groups**
0	21	0.982757	X
1	21	1.26304	X
2	21	1.27257	X
3	21	1.29861	X
4	21	1.31047	X
5	21	1.31076	X
